# Expression and Clinical Significance of Serum 25-OH-D in pregnant women with SCH (Subclinical Hypothyroidism) and GDM (Gestational Diabetes Mellitus)

**DOI:** 10.12669/pjms.345.15719

**Published:** 2018

**Authors:** Xue Zhou, Zhihong Li, Ben Li, Shuqin Guo, Mingyan Yao

**Affiliations:** 1 Xue Zhou, Department of Endocrinology, Baoding First Central Hospital, Baoding 071000, P.R. China; 2 Zhihong Li, Department of Endocrinology, Baoding First Central Hospital, Baoding 071000, P.R. China; 3 Ben Li, Department of Thoracic Surgery, Hebei University Affiliated Hospital, Baoding 071000, P.R. China; 4 Shuqin Guo, Department of Endocrinology, Baoding First Central Hospital, Baoding 071000, P.R. China; 5 Mingyan Yao, Department of Endocrinology, Baoding First Central Hospital, Baoding 071000, P.R. China

**Keywords:** Subclinical hypothyroidism, Gestational diabetes mellitus, Serum 25-OH-D3, Thyroid autoantibody

## Abstract

**Objective:**

To analyze the level and clinical significance of serum 25-hydroxyvitamin D (25-OH-D) levels in pregnant women with subclinical hypothyroidism (SCH) and gestational diabetes mellitus (GDM).

**Method:**

One hundred pregnant women of 24-28 weeks’ pregnancy with SCH combined GDM were selected into the observation group, and 100 healthy pregnant women were selected into the control group during the corresponding period. Examined the thyroid stimulating hormone (TSH), free thyroxine (FT4), 25-OH-D, serum calcium (Ca2+), fasting plasma glucose (FPG), and glycosylated hemoglobin (HbAIc) levels and thyroid peroxide antibody (TPOAb), Thyroglobulin antibody (TgAb) status. Examine and compare TSH, FT4, Ca2+, FPG, HbAIc, TPOAb, and TgAb at different levels of serum 25-OH-D in the observation group. Compared the 25-OH-D levels and the ratio of different contents of 25-OH-D of TPOAb-positive and TgAb-positive SCH pregnant women.

**Result:**

The levels of TSH, FPG and HbAIc in the observation group were significantly higher than those in the control group (P<0.01). Through comparison of FT4 levels between the two groups, the difference had no statistical significance (P>0.05). The levels of 25-OH-D and Ca2+ in the observation group was significantly lower than those in the control group, and the difference had statistical significance (P<0.01). Through comparison of TSH, Ca2+, FPG and HbAIc in groups with different serum 25-OH-D levels, the difference had statistical significance (P<0.01). The positive rates of TPOAb and TgAb of pregnant women in the observation group were higher than in the control group (P<0.05). The vitamin D deficiency rate of TPOAb or TgAb positive pregnant women in the observation group was higher than those in the TPOAb or TgAb negative pregnant women, the difference had statistical significance (P < 0.05).

**Conclusion:**

blood glucose level in pregnant women with GDM and SCH increased significantly, blood calcium level decreased significantly. This group of people are more likely to have VitD deficiency. Thyroid stimulating hormone and blood glucose levels in pregnant women are negatively correlated with VitD levels. Therefore, serum 25-OH-D level can be used as an important reference index for gestational diabetes mellitus with subclinical hypothyroidism, and it has great clinical significance to maintain it at a normal level.

## INTRODUCTION

Subclinical hypothyroidism (SCH) is a common endocrine subclinical disease. TSH level is higher than the upper limit of normal reference during pregnancy, and free thyroxine (FT4) is within the normal reference range. Now with the incidence up to 4% to 10%, it may be combined with metabolic diseases such as hypertension, hyperglycemia, dyslipidemia, etc.^1^-^5^, and attract increasing attention from people. Foreign research findings on subclinical hypothyroidism and vitamin D nutritional status remain controversial. A research by Aljohani et al.^6^ in Saudi Arabia showed that patients with subclinical hypothyroidism have higher vitamin D level than healthy people. Gestational diabetes mellitus (GDM) with normal pre-pregnancy glucose metabolism only occurs during pregnancy. The incidence of gestational diabetes (GDM) has increased significantly in recent years.^7^ GDM pregnant women has abnormal insulin metabolism, as well as other hormone secretion abnormalities, including the highest proportion of thyroid dysfunction.^8^

Recent studies have showed that VitD can participate in the immune regulation process through its active form 1,25(OH)2D3, inhibit the maturation and differentiation of dendritic cells, and also promote the regulatory T lymphocyte proliferation through cytokines, and relate to various autoimmune diseases including sexual diseases, including autoimmune thyroid disease.^9^ Through researching thyroid autoantibodies and VitD content in pregnant women with SCH-GDM, this paper has discussed the relationship between subclinical hypothyroidism, diabetes and VitD levels, and the important significance of 25-OH-D detection.

## METHODS

One Hundred pregnant women with GDM and SCH received by Baoding First Central Hospital from January 2016 to December 2017 were selected as the object of study, as the observation group at the age of 20 to 39 with 24-28 weeks’ gestation. The pregnant women in the observation group met the diagnostic criteria for SCH and GDM. Patients who recently received vitamin D and patients with diabetes and thyroid diseases were excluded. One hundred healthy pregnant women were selected in the same period, as the control group, at the age of 21 to 38, and with 24-28 weeks’ gestation. The difference of comparison between the age of pregnant women and gestation in two group has no statistical significance (P > 0.05), as shown in Table-I.

**Table-I:** Comparison of pregnant women’s general condition in two groups (x±s).

Group	Case No. (n)	Age (Year)	Gestation (W)
Observation group	100	26.70±4.51	25.91±1.21
Control group	100	26.51±4.70	25.80±1.40
t value		0.292	0.594
P value		P>0. 05	P>0. 05

### Methods

Specimens collection and processing (1) Blood glucose specimens collection: pregnant women selected were fasted for 10 H, and 75 g glucose tolerance test (OG-TT) was performed with an empty stomach in the next morning to detect fasting plasma glucose (FPG) and glucose values at one and two hour after glucose intake^2^ 5 ml of fasting venous blood were collected from subjects and serum was separated for measurement at 3000r/min centrifugation for 10 minutes. 25-OH-D, blood calcium (Ca2+), fasting blood glucose (FPG), glycosylated hemoglobin (HbAIc), thyroid peroxidation antibody (TPOAb), thyroglobulin antibody (TgAb) and other indicators of thyroid function were detected.

Instrument and reagent Serum levels of TSH, FT4, and FBG were measured by using Kermankult UniCel Dx1800 automatic chemical immunoassay analyzer; 25-OH-D level was measured by using Roche Elecsys 2010 electro-chemiluminescence auto immunoassay analyzer and kits. The instruments and kits were made by Roche Group. Ca2+ concentration was measured by o-cresolphthalein spectrophotometric method. HbAIc was measure by IMMAGE800 specific protein analyzer made in US. Beckman DXI800 chemiluminescence analyzer 2010 and supplementary reagents were used to measure TPOAb and TgAb.

### Diagnostic criteria

Diagnosis of GDM the blood glucose values of FPG and at one and two hour after glucose intake were 5.1 mmol/L, 10.0 mmol/L, and 8.5 mmol/L, respectively. The blood glucose value at any point meeting or exceeding the above criteria can be diagnosed.^10^

SCH during gestation Based on the diagnostic criteria of subclinical hypothyroidism related to the 2012 China Guidelines for the Diagnosis and Treatment of Thyroid Diseases during Pregnancy and Postpartum^11^: 3mIU/L<TSH<10mIU/L, FT4<12 pmol/L.

### VitD reference standard

Serum 25-OH-D <20 ng/ml means lack of VitD; 20 ng/ml ≤ 25-OH-D <30 ng/ml means VitD deficiency; ≥ 30 ng/ml means appropriate VitD content.^12^

Ca2+ reference standard: Normal range of Ca2+: 0.7~1.3mmol/L

HbAIc reference standard: Normal range of HbAIc: 4~6%

### Statistical Analysis

Statistical analysis was performed by using SPSS22.0 (SPSS Inc, Chicago, IL, USA) software, all data under normality test, numerical variables represented by $$$S, comparison among groups performed by using independent sample t test, compare within groups performed by using paired t test; comparison of categorical variable among groups were performed by using Chi-square ($$$2) test; multiple dataset were compared through variance analysis; Spearman correlations were used for correlation analysis. P<0.05 was considered to have statistical significance.

## RESULTS

Comparison of pregnant women’s relevant indexes in two groups FPG, TSH, and HbAIc levels of pregnant women in the observation group were significantly higher than those in the control group, and the difference had statistical significance (P<0.01). The difference had no statistical significance through comparison of FT4 levels in the two groups (P>0.05). The levels of 25-OH-D and Ca2+ in the observation group were significantly lower than those in the control group, and the difference had statistical significance (P<0.01), as shown in Table-II.

**Table-II:** Comparison of serum levels of TSH, FT4, 25-OH-D, Ca2+, FPG and HbAIc of pregnant women in two groups (*X̄*±S).

Group	Case No. (n)	TSH (mmol/L)	FT4 (pmol/L)	25-OH-D (ng/mL)	Ca^2+^(mmol/L)	FPG (mmol/L)	HbAIc (%)
Observation group	100	7.25±1.54	7.83±4.05	27.86±7.35	0.88±0.13	6.75±1.84	7.16±1.89
Control group	100	1.75±0.48	7.72±3.95	39.25±8.90	1.15±0.23	4.81±1.53	4.84±1.50
t value		34.096	0.194	-9.868	-10.220	8.107	9.615
P value		P<0.01	P>0.05	P<0.01	P<0.01	P<0.01	P<0.01

Comparison of relative indexes of different levels of 25-OH-D in the observation group The difference through the comparison of FPG, TSH, Ca2+ and HbAIc at different levels of 25-OH-D had statistical significance (P<0.01). Through comparison of FT4 levels in each group, the difference had no statistical significance (P> 0.05), as shown in Table-III.

**Table-III:** Comparison of thyroid and blood glucose levels measured at different levels of 25-OH-D in observation group (*X̄*±S).

25-OH-D (ng/mL)	Case No. (n)	TSH (mmol/L)	FT4 (pmol/L)	Ca2+ (mmol/L)	FPG (mmol/L)	HbAIc (%)
Deficient group (<20)	11	9.82±2.03	7.72±4.05	0.73±0.09	7.93±1.85	7.88±1.83
Insufficient group (20~30)	64	7.96±1.89	7.87±4.35	0.82±0.12	7.04±1.23	7.10±1.01
Sufficient group (≥30)	25	3.34±1.50	7.99±4.60	1.05±0.23	6.41±1.52	6.40±1.50
F value		17.78	0.540	4.728	10.17	8.152
P value		P<0.01	P>0.05	P<0.05	P<0.01	P<0.01

Comparison of positive rates of thyroid autoantibodies of pregnant women in two groups The positive rates of TPOAb and TgAb of pregnant women in the observation group were higher than those in the control group, and the difference had statistical significance (P<0.05), as shown in Table-IV.

**Table-IV:** Comparison of positive rates of thyroid autoantibodies of pregnant women in two groups [n (%)].

Group	Case No.	TPOAb (+)	TgAb (+)
Observation group	100	27 (27.0)	23 (23.0)
Control group	100	11(11.0)	10 (10.0)
^2^ value		8.317	6.133
P value		P<0.01	P<0.05

Relationship between thyroid autoantibodies and 25-OH-D of pregnant women in the observation group pregnant women with TPOAb or TgAb in the observation group have higher rate of vitamin D deficiency than that in TPOAb or TgAb negative women. The difference was statistical significance (P<0.05), as shown in Table-V.

**Table-V:** Comparison of thyroid autoantibody at same level of 25-OH-D in observation group [n (%)].

25-OH-D (ng/mL)	TPOAb				TgAb			

	Positive	Negative	^2^ value	P value	Positive	Negative	^2^ value	P value
Case No.	27	73			23	77		
Deficient group (<20)	15 (55.56)	25 (34.25)	6.860	P<0.05	13 (56.52)	24 (31.17)	7.328	P<0.05
Insufficient group (20~30)	10 (37.04)	25 (34.25)	2.225	P>0.05	8 (34.78)	26 (33.77)	2.070	P>0.05
Sufficient group (≥30)	2 (7.41)	23(31.51)	1.026	P>0.05	2 (8.70)	27 (35.06)	0.577	P>0.05

## DISCUSSIONS

SCH is a common abnormal thyroid dysfunction during pregnancy with an increasing trend year by year^13^ which can directly affect the entire pregnancy process as one of the high risk factors for preterm birth and miscarriage. GDM is a common and frequently occurring disease in obstetrics which mostly occurs in the middle and later stages of pregnancy. The risk of complications during pregnancy increases significantly in GDM pregnant women. The morbidity of neonatal fetus growth under limitation, fetal macrosomia, miscarriage, premature birth, fetal malformations, neonatal respiratory distress syndrome, and neonatal hypoglycemia has significantly increased.^14^ Pregnancy with SCH may affect the descendant intelligence development, increase miscarriage, premature delivery, fetal growth restriction, low birth weight infants, fetal distress, and placental abruption, premature rupture of membranes, and postpartum hemorrhage^15^ and lead to other adverse pregnancy outcomes. VitD can affect the proliferation, differentiation and apoptosis of immune cells and regulating the function of the immune system through binding with its receptors.^16^ The VitD level in human body can be assessed clinically by measuring serum 25-OH-D levels, which can reflect the total VitD level including those from the sun and the diet, as well as those from liver conversion.^17^,^18^ Studies have shown that the demand for VitD during pregnancy is more than two times higher than in non-pregnant period. Therefore, VitD deficiency during pregnancy has become a worldwide phenomenon.^19^ The morbidity of VitD deficiency in China is also high.^20^ 25-OH-D is the intermediate metabolite of VitD, with long half-life period and stable expression in blood. Therefore, detection of VitD concentration in human body can be used to measure its concentration.^21^ Studies confirm that 25-OH-D in the blood through binding with VitD receptor in the nucleus can regulate cell expression, and thereby prevent cell damage.^22^ There is research confirmed that serum 25-OH-D levels in pregnant women after being given sufficient vitamin D supplementation increased significantly, while insulin resistance significantly alleviated.^23^ Meanwhile, it has been confirmed that serum 25-OH-D levels in patients with hypothyroidism was significantly lower than those in normal control group.^24^ US national survey and research report shows that there exists a positive correlation between TPOAb and SCH prevalence, and TPOAb positive patients are closely related to the occurrence of clinical autoimmune diseases.^25^

It can be found in this study that levels of TSH, FPG and HbAIc in GDM and SCH pregnant women were significantly higher than those in normal pregnant group; the levels of 25-OH-D and Ca2+ in GDM and SCH pregnant women were significantly lower than those in normal pregnant group. With the increase of 25-OH-D level, through the comparison of levels of FPG, TSH, and Ca2+ in different groups, the levels of FPG, TSH, and HbAIc in the 25-OH-D deficient group were lower than those in the 25-OH-D sufficient group. However, Ca2+ levels reduced compared with those in 25-OH-D deficient group and sufficient group. The positive rates of TPOAb and TgAb in GDM and SCH pregnant women were higher than normal pregnant women. Pregnant women with TPOAb or TgAb positive have a higher rate of VitD deficiency than TPOAb or TgAb negative pregnant women. When the serum 25-OH-D of GDM and SCH pregnant women is at a low level, it may cause humoral immune function disorders, and increase the demand for VitD, to result in 25-OH-D level decline and VitD deficiency. It is the current situation that GDM and SCH pregnant women have VitD deficiency problem. Therefore, serum 25-OH-D can be used as an important monitoring index for GDM and SCH.

To sum up, VitD deficiency in pregnant women will affect the health of pregnant women and inevitably affect the level of fetal VitD. As the level and storage of fetal VitD are determined by the level of VitD in pregnant women, VitD deficiency in pregnant women may result in low levels of VitD in fetuses and postnatal newborns.^26^ GDM and SCH pregnant women have higher VitD deficiency occurrence than normal pregnant women. Thyroid stimulating hormone and blood glucose levels in pregnant women are negatively correlated with VitD levels. While receiving retreatment, GDM and SCH pregnant women should actively replenish VitD to protect maternal and child health. The sample size of this study is limited, and the specific mechanism has not been discussed in depth. It is still to be further studied. In addition, whether the appropriate supplement of VitD can reduce the incidence of SCH needs further study.

### Author’s Contributions

***XZ, ZL:*** Designed this study and prepared this manuscript.

***BL, SG:*** Performed this study.

***MY:*** Collected and analyzed clinical data.
